# Psychological and Demographic Determinants of Substance Use and Mental Health During the COVID-19 Pandemic

**DOI:** 10.3389/fpubh.2021.680028

**Published:** 2021-06-25

**Authors:** Fatima Mougharbel, Hugues Sampasa-Kanyinga, Brandon Heidinger, Kim Corace, Hayley A. Hamilton, Gary S. Goldfield

**Affiliations:** ^1^Interdisciplinary School of Health Sciences, University of Ottawa, Ottawa, ON, Canada; ^2^Healthy Active Living and Obesity Research Group, Children's Hospital of Eastern Ontario Research Institute, Ottawa, ON, Canada; ^3^School of Epidemiology and Public Health, University of Ottawa, Ottawa, ON, Canada; ^4^The Royal's Institute of Mental Health Research, Ottawa, ON, Canada; ^5^Faculty of Medicine, University of Ottawa, Ottawa, ON, Canada; ^6^The Royal Ottawa Mental Health Centre, Ottawa, ON, Canada; ^7^Centre for Addiction and Mental Health, Institute for Mental Health Policy Research, Toronto, ON, Canada; ^8^Centre for Addiction and Mental Health, Campbell Family Mental Health Research Institute, Toronto, ON, Canada; ^9^Dalla Lana School of Public Health, University of Toronto, Toronto, ON, Canada; ^10^Department of Pediatrics, University of Ottawa, Ottawa, ON, Canada

**Keywords:** alcohol use and alcohol problems, binge drinking, anxiety, depression, concurrency, COVID-19

## Abstract

**Background:** Alcohol consumption and distress have increased among Canadians since the start of the COVID-19 pandemic.

**Methods:** We examined whether sociodemographic and COVID-19-related worries are associated with various combinations of alcohol consumption and comorbid psychological distress variables among a Canadian sample of adults. Data were derived from a sample of Canadian adults (*N* = 1,005, 49.6% female) who participated in an online survey in May 2020. Four multivariate ordinal logistic regression models were used to estimate the odds of binge drinking, increased alcohol consumption during the pandemic, and psychological distress. Predictor variables used in the analyses included self-reported sociodemographic characteristics, financial worries, COVID-19 impact on work, and worrying about getting ill.

**Results:** Women were found to have higher odds of increased drinking and anxiety. Also being divorced, separated, or widowed was associated with higher odds of binge drinking and anxiety, and binge drinking and depression. Furthermore, being 60 or older was associated with lower odds of binge drinking and depression and increased drinking and depression, as well as lower odds of increased drinking and depression and increased drinking and anxiety. High income groups were associated with higher odds of binge drinking, increased drinking, and mental distress. Compared to those less worried, being very worried about finances were associated with higher odds of binge drinking and anxiety, increased drinking and anxiety, and increased drinking and depression. Also, being very worried about getting ill with COVID was associated with higher odds of binge drinking and anxiety and increased drinking and anxiety.

**Conclusion:** Our findings identify several demographic and COVID-related worries for increased odds of alcohol intake and co-morbid psychological distress during the COVID-19 pandemic, including identifying as a woman, high income groups, being divorced, separated or widowed, and experiencing financial worries and COVID illness worries. These characteristics should be considered when developing prevention and treatment programs for adults with problematic alcohol use and comorbid anxiety and depression.

## Background

At the beginning of Spring 2020, the coronavirus disease 2019 (COVID-19) hit many countries globally, including Canada. This led the federal and provincial authorities to implement stringent measures to limit the virus' spread and flatten the curve. These measures included closures of schools, non-essential businesses, and community centers, resulting in most of the population being confined to their homes. Upon a phased reopening, strict physical distancing measures were implemented, resulting in diminished frequency and quality of social interactions (1).

In no time in recent human history have individuals been subjected to such social and occupational restrictions, making the current COVID-19 environment uniquely stressful. The COVID-19 pandemic, similar to other pandemics, placed individuals at an increased risk for developing (or exacerbating) anxiety, depression, and problematic substance use as the demands of coping with the pandemic may outweigh perceived emotional and financial resources to cope (2). Indeed, recent studies linked this pandemic and its associated consequences with increased rates of depression and anxiety (3–6). For example, a survey conducted during the COVID-19 pandemic (June 2020) among 5,470 adults in the USA showed that the percentage reporting symptoms of anxiety was three times higher than what was reported in 2019 (25.5 vs. 8.1%), and the percentage reporting symptoms of depression was about four times higher than in 2019 (24.3 vs. 6.5%) (3).

Several studies have examined substance use, such as alcohol consumption, during the COVID-19 pandemic (7–9). A recent study in the USA (9) among 1,540 adults examined changes in alcohol consumption from before to during the COVID-19 pandemic through the number of days of alcohol use and binge drinking over the past 30 days. This study showed an increase of 14% in the frequency of alcohol consumption, and an increase of 19% in binge drinking during the pandemic. Interestingly, women in this study showed a significant increase of 41% in binge drinking during the pandemic. A Canadian survey of 1,009 adults found that one in five Canadians who stayed at home more during the pandemic reported an increase in both quantity and frequency of alcohol consumption. Boredom, stress and changes in routine were among the strongest contributing factors for increased alcohol consumption reported in this survey (7).

Increases in alcohol consumption and binge drinking, relative to pre-pandemic levels, could be due to many factors including a maladaptive way of coping with stress and anxiety (i.e., self-medicating) (10, 11). Additionally, this pandemic has increased feelings of social isolation and loneliness, which are well-known to increase the risk of problematic alcohol consumption (12–14). This is concerning because alcohol can suppress immune function (15, 16), consequently leaving individuals more vulnerable to COVID-19, respiratory illnesses (16), systemic inflammation (17, 18), and a range of other disease states that can lead to hospitalization (19). Moreover, problematic alcohol use increases the risk of metabolic disorders (20), cardiovascular disease (21–23), neurocognitive disorders (24), mental illness (25), and all cause mortality (26).

Although, the determinants of anxiety, depression, and problematic substance use are each widely studied, less attention has been paid to the factors associated with experiencing both mental health and substance use problems at the same time, a phenomenon known as concurrency. It can also be called, dual diagnosis, dual disorders, or co-occurring substance use and mental health problems (27). Previous data indicated an association between concurrent disorders and high costs of care, and increased morbidity and mortality rate (28–30). People with concurrency experience poorer physical (31) and mental health (32) than individuals with a single problem. They also have higher use of medical and psychological health services which place a high economic burden on the health care system (29, 33–35).

The high prevalence and personal and economic costs of concurrency highlight a crucial need for a better understanding of its determinants, and this is especially important during the pandemic to inform the development of effective treatment and prevention strategies. The relationship between the two concurrent conditions is complex (36) and factors that contribute to this complexity can include demographic and individual factors, but it also can be social-cultural in nature (8, 37). While some studies examined the impact of the COVID-19 pandemic on mental health and aspects of substance use such as substance use disorders (38) and the impact of this association on suicide (39), no studies, to our knowledge have investigated the determinants of concurrency during the pandemic. In addition, factors relating to the social determinants of health such as marital status, socio-economic status, changes to employment status, or being a parent of young children have not yet been examined.

Accordingly, the purpose of this study was to examine the association between socio-demographic, and COVID-19 related worries and combinations of concurrent alcohol use, depression, and anxiety in a sample of Canadian adults during the COVID-19 pandemic.

## Methods

### Study Sample

This study performed a secondary data analysis based on data from a survey conducted by the Centre for Addiction and Mental Health in collaboration with Delvinia, a research technology and market survey company. An English-speaking Canadian adult sample (*N* = 1,005) completed an online survey in the early part of the pandemic from May 8 to 12, 2020, through an automated web-based panel which links organizations to real people to obtain data (http://www.delvinia.com/solutions/askingcanadians/, accessed August 18, 2020). Participants were sourced from the AskingCanadians web panel (http://corporate.askingcanadians.com/) of over 1 million Canadians hosted by Delvinia (data are publicly available: http://www.delvinia.com/camh-coronavirus-mental-health/). Respondents were recruited on the basis of loyalty collaborations with retailers, departmental stores, and airlines. The study was approved by the Research Ethics Board at the Centre for Addiction and Mental Health. Also, this study has been reviewed by the Research Ethics Boards of the Children's Hospital of Eastern Ontario (CHEO). Participation was voluntary, and all participants provided their consent electronically. Quota sampling was utilized such that the sample of 1,005 adult respondents reflected the distribution of English-speaking Canadians by gender, age, and geographic region (British Columbia, Alberta, Saskatchewan/Manitoba, Ontario, Quebec/Atlantic provinces, and the territories including Yukon, Nunavut, and the Northwest Territories).

### Distress Variables Measures

#### Depressive Symptoms

Depressive symptoms were assessed using a 3-item short form of the Center for Epidemiologic Studies Depression Scale (CES-D) (40). The 3-item scale asks participants how frequently they felt depressed, lonely, or hopeful about the future over the past 7 days. Items were scored using a 4-point Likert-type frequency scale from “1 = Rarely or none of the time (<1 day)” to “4 = Most or all of the time (5–7 days).” The item on feeling hopeful was reverse scored, so higher scores reflect less hope about the future. A composite score was calculated, with raw scores ranging from 3 to 12. For data analyses, participants who scored ≥10 were classified as having higher severity of symptoms, while participants scoring <10 were considered to be less symptomatic (40).

#### Anxiety

Anxiety was measured using the Generalized Anxiety Disorder (GAD-7) scale (41). This is a self-report measure that includes seven items; for each one, participants were asked to rate the frequency in which they experienced anxiety symptoms over the last 2 weeks on a Likert-type frequency scale from 0 = “Not at all” to 3 = “Nearly every day” (41). A composite score was calculated, with raw scores ranging from 0 to 21. Individuals who scored 0–9 were considered as having no to mild anxiety symptoms, and those who scored ≥10 were classified as having symptoms of moderate to severe anxiety (42).

### Substance Use Measures

#### Increased Drinking

Increased alcohol consumption was measured using the following item: “In the PAST 7 DAYS, did you drink more ALCOHOL, about the same, or less alcohol overall than you did before the COVID-19 pandemic started?” Response options were coded 1 = “Drink much more alcohol,” 2 = “Drink slightly more alcohol,” 3 = “No change,” 4 = “Drink slightly less alcohol,” 5 = “Drink much less alcohol.” A dichotomous measure was created to reflect increased alcohol consumption (drink much more or slightly more alcohol) or not. Individuals who did not report consuming alcohol in the past week were coded as no increase in use.

#### Binge Drinking

Binge drinking was assessed by the item: “how many of the PAST 7 DAYS did you drink [four (if woman) or five (if man) or five (if another gender)] or more drinks on one occasion? A drink means 341 ml or 12 oz. bottle of beer or cider/cooler (5% alcohol content), a 142 ml or 5 oz. glass of wine (12% alcohol content), or a straight or mixed drink with 43 ml or 1.5 oz. of liquor (40% alcohol content – e.g., rye, gin, rum).” Response options ranged from 0 to 7 days. In the data analysis, binge drinking was operationally defined as consuming five or more drinks per episode for men and as four or more drinks per episode for women. This was coded dichotomously as binge drinking (1) or not (0). Individuals who did not report consuming alcohol in the past week were coded as not binge drinking.

#### Dependent Variables - Increased Alcohol Consumption and Distress (Concurrency Variables)

Risk variables were created using the following combinations of both distress and problematic drinking variables: (1) binge drinking and anxiety, (2) binge drinking and depression, (3) increased drinking and anxiety, (4) increased drinking and depression. Risk items were coded from 0 to 2 with 0 = no risk and 2 = 2 risks for each combination of mental distress and alcohol use variables.

#### Independent Variables - COVID-19 Relevant Factors

For COVID-19 illness worries and financial worries, participants were asked the following questions: (1) Whether they are worried that they or someone close to them will get ill from COVID-19? (2) Whether they are worried about the impact of COVID-19 on their personal financial situation? Responses included (1 = very worried; 2 = somewhat worried; 3 = not very worried; 4 = not at all worried). COVID-19 illness worries, and financial worries variables were coded from 0 to 3 after reversing the scoring (0 = not very or not at all worried; 1 = somewhat worried; 2 = very worried).

#### Sociodemographic Factors

Sociodemographic measures consisted of the following variables including how they were coded in the analysis:

Gender (1 = man, 2 = woman, 3 = other); age (1 = 18–39, 2 = 40–59, 3 = 60+ years), marital status (1 = married or living with a partner; 2 = divorced, separated, or widowed; 3 = never married), education (1 = not completed high school; 2 = completed high school; completed some post-secondary graduation; 3 = completed college or university education), number of children aged 6–12 years at home, and household income (1 = < $40,000; 2 = $40,000–$79,000; 3 = $80,000–$119,000; 4 = $120,000 or more). Ethnicity included (Asian – East; Asian – South; Asian – southeast; Black; Indigenous; Latin American; Middle Eastern; White; Mixed heritage; Other), we dichotomized this variable as the following (0 = white; 1 = other).

### Statistical Analysis

Analyses were done within SPSS (version 26) and Stata 14.1 (Stata Corporation, College Station, TX, USA). Descriptive analysis was used to describe the sample. Correlations were calculated to assess relations and directionality between independent variables (age, gender, marital status, education, number of children, income, ethnicity, financial worries, COVID-19 illness worries, and COVID-19 impact on working hours) and primary dependent variables (binge drinking and anxiety, binge drinking and depression, increase drinking and anxiety, increase drinking and depression). Multivariate ordinal logistic regression was performed for all candidate predictor variables to examine their associations with co-occurrence of (1) binge drinking and anxiety, (2) binge drinking and depression, (3) increase drinking and anxiety, and (4) increase drinking and depression. Odds ratios (OR) and 95 % confidence intervals (CI) for fully adjusted models are presented.

## Results

### Demographics and Characteristics of the Study Variables

Table 1 presents the characteristics of the full sample for all variables of interest. Seventy-one percent of the study sample was comprised of white participants. Approximately 61.8% of the participants were married or living with a partner, 9.1% had at least one child aged 6–12 years, and 72.9% had completed college/university. Furthermore, most of the study participants (77.2%) were worried about them or someone close to them getting ill from COVID-19, and 69.2% of participants had financial worries.

**Table 1:** Descriptive characteristics of the study variables.

	**Frequency**	**Valid percent**
Gender (*n* = 1,002)		
Male	504	50.1%
Female	498	49.6%
Other	3	0.3%
Age (*n* = 1,005)		
18–39 years	394	39.3%
40–59 years	306	30.4%
60^+^ years	305	30.3%
Ethnicity (*n* = 985)		
White	698	71%
Others	287	29%
Income (*n* = 839)		
<40 k	128	15.3%
40–79 k	268	31.9%
80–119 k	226	26.9%
120 K^+^	217	25.9%
Education (*n* = 998)		
Not completed high school	19	1.9%
High school/some level of post-secondary school	251	25.9%
College or University graduate	728	72.9%
Number of children/young children (6–12) (*n* = 1,005)		
0	914	90.9%
1	61	6.1%
2	28	2.8%
3	2	0.2%
Marital status (*n* = 992)		
Married or living with a partner	613	61.8%
Divorced, separated, or widowed	128	12.9%
Not married	251	25.3%
COVID illness worries (*n* = 1,005)		
Not very/not at all worried	229	22.8%
Somewhat worried	511	50.8%
Very worried	265	26.4%
Financial worries (*n* = 1,005)		
Not very/not at all worried	310	30.8.%
Somewhat worried	475	47.3%
Very worried	220	21.9%
Depression scores (*n* = 1,005)		
No depression to mild depression (3–9)	930	92.5%
Moderately severe depression (10–12)	75	7.5%
Anxiety score (*n* = 1,005)		
No to mild anxiety (0–9)	749	74.5%
Moderate to severe anxiety (10–21)	256	25.5%
Binge drinking (*n* = 1,003)		
Yes	238	23.7%
No	765	76.3%
Increase drinking (*n* = 1,005)		
Yes	253	25.2%
No	752	74.8%
Binge drinking and Anxiety^a^ (*n* = 1,003)		
0	606	60.4%
1	300	29.9%
2	97	9.7%
Binge drinking and depression (*n* = 1,003)		
0	712	71%
1	269	26.8%
2	22	2.2%
Increase drinking and anxiety (*n* = 1,005)		
0	595	59.2%
1	311	30.8%
2	99	10%
Increase drinking and depression (*n* = 1,005)		
0	703	70.0%
1	276	27.5%
2	26	2.5%

*Outcome variables were coded from 0 to 2 with 0 = no risk; 1= risk for either mental distress or alcohol consumption; and 2 = 2 risks for each combination of mental distress and alcohol use variables*.

Furthermore, 7.5% of participants had elevated depressive symptoms and 25.5% experienced moderate to severe anxiety. Furthermore, 23.7% reported binge drinking and 25.2% reported an increase in alcohol consumption. Concurrency variables indicated that higher percentages of respondents reported binge drinking and anxiety (9.7%) and increased drinking with anxiety (10%) compared to binge drinking and depression (2.2%) and increased drinking and depression (2.5%).

Bivariate correlations for all variables of interest are shown in Table 2. Age was inversely correlated with binge drinking and anxiety (r = −0.20, *p* < 0.01), and binge drinking and depression (r = −0.16, *p* < 0.01), as well as increased drinking and anxiety (r = −0.16, *p* < 0.01), and increased drinking and depression (r = −0.11, *p* < 0.01).

**Table 2:** Spearman correlations between all main study variables.

	**1**.	**2**.	**3**.	**4**.	**5**.	**6**.	**7**.	**8**.	**9**.	**10**.	**11**.	**12**.	**13**.	**14**.	**15**.	**16**.	**17**.
1. Age	-																
2. Gender	0.008	-															
3. Ethnicity	−0.283^**^	−0.091^**^	-														
4. Income	−0.109^**^	−0.070	0.010	-													
5. Education	−0.118^**^	−0.040	0.097^**^	0.220^**^	-												
6. Having young children (6–12)	−0.127^**^	0.006	0.088^**^	0.051	0.045	-											
7. Marital status	−0.216^**^	0.041	0.084^**^	−0.315^**^	−0.100^**^	−0.120^**^	-										
8. Covid illness worries	0.059	0.123^**^	−0.180^**^	−0.031	0.080^*^	0.050	−0.040	-									
9. Financial worries	−0.066^*^	0.064^*^	0.190^**^	−0.100^**^	0.070^*^	0.050	0.010	0.450^**^	-								
10. Moderate to severe anxiety	−0.137^**^	0.100^**^	0.100^**^	−0.031	−0.033	0.054	0.008	0.324^**^	0.352^**^	-							
11. Moderate to severe depression	−0.048	0.104^**^	−0.002	−0.126^*^	−0.041	0.100^*^	0.121^**^	0.140^**^	0.160^**^	0.400^**^	-						
12. Binge drinking	−0.158^**^	−0.100^**^	0.052	0.080^*^	−0.041	0.100^*^	0.012	0.070^*^	0.080^*^	0.200^**^	0.037	-					
13. Increase drinking	−0.112^*^	0.029	−0.042	0.172^*^	0.050	0.040	−0.042	0.020	0.019	0.182^**^	0.062^*^	0.475^**^	-				
14. Binge drinking and Anxiety	−0.200^**^	0.009	0.100	0.033	−0.051	0.100^*^	0.008	0.253^**^	0.300^**^	0.770^**^	0.287^**^	0.745^**^	0.387^**^	-			
15. Binge drinking and depression	−0.162^**^	−0.026	0.043	0.007	−0.051	0.100^*^	0.080^*^	0.130^**^	0.144^**^	0.350^**^	0.482^**^	0.875^**^	0.429^**^	0.800^**^	-		
16. Increase drinking and anxiety	−0.163^**^	0.100^**^	0.040	0.100^**^	0.012	0.053	−0.020	0.224^**^	0.240^**^	0.756^**^	0.300^**^	0.400^**^	0.752^**^	0.743^**^	0.500^**^	-	
17. Increase drinking and depression	−0.117^**^	0.100^*^	−0.042	0.100^*^	0.031	0.052	0.024	0.100^*^	0.100^*^	0.322^**^	0.480^**^	0.419^**^	0.900^**^	0.460^**^	0.600^**^	0.800^**^	-

* *p < 0.05*,

** *p < 0.01*.

*Gender (male/female), ethnicity (white/non-white), income (<40,000, 40,000–79,000, 80,000–120,000, >120,000 and more), education (less than high school; completed high school or some post-secondary graduation; college or university), marital status (married or living with a partner; widowed, divorced, separated; or never married), COVID illness worries (not very or not al all worried; somewhat worried; very worried); COVID financial worries (not very or not al all worried; somewhat worried; very worried)*.

Being a woman was correlated with increased drinking and anxiety (r = 0.10, *p* < 0.01), and increased drinking and depression (r = 0.10, *p* < 0.05). Income was positively correlated with increased drinking and anxiety (r = 0.10, *p* < 0.01) and increased drinking and depression (r = 0.10, *p* < 0.05). Having elementary school age children 6–12 years was positively associated with binge drinking and anxiety (r = 0.10, *p* < 0.05) and binge drinking and depression (r = 0.10, *p* < 0.05), but not increased drinking and distress. Furthermore, being divorced, separated, or widowed was positively associated with binge drinking and depression (r = 0.10, *p* < 0.05). Worrying about getting ill from COVID-19 was positively associated with binge drinking and anxiety (r = 0.25, *p* < 0.01), and binge drinking and depression (r = 0.13, *p* < 0.01), and increased drinking and anxiety (r = 0.22, *p* < 0.01), and depression as well (r = 0.10, *p* < 0.05). Moreover, financial worrying was positively associated with binge drinking and anxiety, and depression (r = 0.30, *p* < 0.01) and binge drinking and depression (r= 0.14, *p* < 0.01), as well as increased drinking and anxiety (r = 0.24, *p* < 0.01), and increased drinking and depression r = 0.10, *p* < 0.05).

### Multivariate Ordinal Regression Modeling of Effects of COVID-19 Risk Factors on Binge Drinking and Increased Drinking and Mental Distress

Results of multivariate ordinal regression analysis examining the association between combinations of distress and alcohol use variables are summarized in Table 3. Results indicate that being older in age was associated with lower odds of binge drinking and anxiety (OR 0.33, 95% CI, 0.22–0.49), and binge drinking and depression (OR 0.39, 95% CI, 0.25–0.61), as well as lower odds of increased drinking and anxiety (OR 0.44, 95% CI, 0.30–0.65), and increased drinking and depression (OR 0.56, 95% CI, 0.37–0.85). In addition to that, identifying as woman was significantly associated with greater odds of increased drinking and anxiety (OR 1.36, 95% CI, 1.03–1.80). Also, being divorced, separated, or widowed was associated with higher odds of binge drinking and anxiety (OR 1.74; 95% CI 1.03–2.92) and binge drinking and depression (OR 1.87; 95% CI 1.10–3.21).

**Table 3:** Multivariate analysis for the associations between all predictor variables and binge drinking and anxiety; binge drinking and depression; increase drinking and anxiety; and increase drinking and depression.

	**Binge drinking and anxiety**	**Binge drinking and depression**	**Increase drinking and anxiety**	**Increase drinking and depression**
	***n* = 836**	***n* = 836**	***n* = 836**	***n* = 836**
	**OR (95% CI)**	**OR (95% CI)**	**OR (95% CI)**	**OR (95% CI)**
Age^a^				
40–59 years	0.80 (0.57–1.13)	0.83 (0.58–1.19)	0.73 (0.52–1.02)	0.76 (0.53–1.10)
60^+^ years	0.33 (0.22–0.49)^***^	0.39 (0.25–0.61)^***^	0.44 (0.30–0.65)^***^	0.56 (0.37–0.85)^**^
Gender^b^				
Female	0.92 (0.70–1.22)	0.84 (0.62–1.13)	1.36 (1.03–1.80) ^*^	1.33 (0.98–1.81)
Income^c^				
40–79 k	1.27 (0.80–2.02)	1.30 (0.79–2.13)	1.48 (0.93–2.36)	1.78 (1.06–2.98) ^**^
80–119 k	1.52 (0.93–2.46)	1.49 (0.88–2.50)	2.17 (1.33–3.52) ^***^	2.44 (1.42–4.20) ^**^
120 k+	1.40 (0.85–2.30)	1.34 (0.78–2.29)	2.12 (1.29–3.49) ^***^	2.38 (1.37–4.14) ^**^
Having young children (6–12)	1.05 (0.74–1.49)	1.19 (0.82–1.71)	1.14 (0.81–1.61)	1.33 (0.93–1.89)
Marital status^d^				
Married	1.09 (0.76–1.57)	0.80 (0.54–1.17)	1.12 (0.78–1.60)	0.87 (0.59–1.27)
Divorced, widowed, separated	1.74 (1.03–2.92)^*^	1.87 (1.10–3.21)^*^	1.41 (0.84–2.35)	1.47 (0.85–2.53)
Covid illness worries^e^				
Somewhat worried	1.22 (0.83–1.79)	0.94 (0.62–1.41)	1.22 (0.83–1.76)	0.90 (0.61–1.33)
Very worried	2.65 (1.68–4.16) ^***^	1.53 (0.95–2.48)	2.36 (1.52–3.69) ^***^	1.25 (0.78–2.01)
Financial worries^f^				
Somewhat worried	1.83 (1.27–2.63) ^***^	1.53 (1.05–2.26) ^**^	1.33 (0.94–1.98)	1.01 (0.70–1.47)
Very worried	3.09 (1.97–4.85) ^***^	1.81 (1.12–2.94) ^**^	2.58 (1.66–4.00) ^***^	1.44 (0.90–2.29)

*OR, odds ratio; CI, confidence interval*.

* *p < 0.05*;

** *p < 0.01*;

*** *p < 0.001*.

*Reference categories:*

a *18–39 years*;

b *male*;

c *<40 k*;

d *Never married*;

e *not at all worried*;

f *not at all worried*.

Furthermore, higher income groups were associated with higher odds of increased drinking and anxiety (OR 2.12; 95% CI, 1.29–3.49), and increased drinking and depression (OR 2.38; 95% CI, 1.37–4.14) compared to low-income groups. In addition to that, experiencing a lot of financial worries was associated with significantly higher odds of binge drinking and anxiety (OR 3.09; 95% CI, 1.97–4.85), and binge drinking and depression (OR 1.81; 95% CI, 1.12–2.94) as well as increased drinking and anxiety (OR 2.58; 95% CI, 1.66–4.00) compared to not being worried. Also, being very worried about getting ill from COVID-19 was associated higher odds of binge drinking and anxiety (OR 2.65; 95% CI, 1.68–4.16), and increased drinking and anxiety (OR 2.36; 95% CI, 1.52–3.69) compared to not being worried about COVID-19 illness.

## Discussion

To our knowledge, this study is the first to examine factors influencing the concurrency of alcohol use and mental health in a Canadian population of adults during the COVID-19 pandemic. Our study identified several sociodemographic, and COVID-19 related worries that were associated with concurrency. Being older in age (60 years and more) was associated with higher odds of binge drinking and distress and increased drinking and distress symptoms. Also, being a woman was associated with higher odds of increased drinking and anxiety. Being divorced, separated, or widowed was also associated with both binge drinking and anxiety and binge drinking and depression. High income was associated with higher odds of binge drinking and anxiety and binge drinking and depression. In addition to that, those who were very worried financially and were very worried about getting COVID-19 illness were more likely to consume alcohol and experience more mental health issues compared to those who worry less, in a dose-response gradient.

As the present study contributed to the literature with an aim to examine COVID-19 factors on symptoms of concurrency, drawing on previous literature examining the pandemic's impact on alcohol consumption and mental health issues separately is informative (9, 43, 44). For example, our study found that that 25.2% of the total sample reported increased alcohol intake during COVID, which is somewhat consistent with the 19% reported from an epidemiological sample of American adults during the pandemic (9), and with the Canadian Centre on Substance Use and Addiction survey which indicated a 20% increase in alcohol consumption among staying at home Canadians because of the pandemic (7). Additionally, our findings indicate that 33 % reported elevated symptoms of anxiety or depression during the pandemic, consistent with a recent meta-analytic review showing a heightened prevalence of psychological problems in association with the COVID-19 pandemic (44).

While previous studies have focused on either mental health or substance use during the pandemic, our study has more specifically examined risk factors for the concurrency of alcohol use and elevated symptoms of anxiety and depression in adults. We found that those who are very worried about the impact of COVID-19 on their finances were more likely to binge or increase drinking and experience feelings of depression and/or anxiety. This would suggest that financial stress related to possibly losing their job or possibly to reduced work hours may lead to increased alcohol intake and heightened distress, perhaps due to maladaptive coping skills. This study also found that being very worried about getting ill from COVID-19 was also associated with higher odds of binge drinking and anxiety, and increased drinking and anxiety compared to those who were less worried. These findings are in line with previous studies examining stress and drinking showing that elevated stress may be associated with increased alcohol consumption (45, 46) as a maladaptive approach to cope with stress and worry.

Moreover, individuals with higher income were more likely to consume alcohol and suffer from mental distress. However, our bivariate analysis indicates that higher income is associated with increased drinking but not mental distress. Thus, income may be a predictor of concurrency more because of boredom and access and not as a coping mechanism for anxiety as higher income individuals are less worried financially and their distress levels are lower. This aligns with previous studies which indicated that people with a higher income reported consuming more alcohol during the pandemic than those of the lower income (47–51). This finding is consistent with behavior economics theory, an approach that explores conditions and factors influencing the consumption of commodities (52, 53) including alcohol intake (54), although, concurrent mental illness has not been examined in these models. According to this approach, more affordability, and few restrictions to access to substances are among the strongest predictors of consumption (54). Thus, limitations in affordability, in turn, limit access to alcohol consumption, which may explain the lower odds of alcohol consumption among lower income groups compared to higher incomes who experienced less financial worries and might have more disposable income.

Our study also showed that older adults were at lower odds of increased alcohol consumption and comorbid psychological distress, consistent with recent literature which examined the impact of COVID-19 relevant factors on mental health, and/or substance use separately (3, 55–57). Canadians 60 years of age and older were less likely to experience concurrency symptoms compared to Canadians 40–59, and 18–39 years. These associations may be attributed to older adults being better able to cope and be more resilient in crisis due to biological stress response, personality attributes, life experience, as well as a better financial stability, social and economic status (58). Furthermore, getting older is associated with enhanced life experience, which may enhance emotional regulation and self compassion, and higher spiritual components of wisdom (59) which can help in coping with crisis.

Our findings showed that identifying as woman was associated with increased odds of increased alcohol and anxiety.

This aligns with previous studies which indicated that women were more likely to experience stress, anxiety and depressive symptoms during the pandemic than men (43, 60, 61). Furthermore, evidence indicated that alcohol consumption increased during the pandemic among women (50, 62). The lockdown added a lot of mental burden on women (60, 63, 64). This can be due to increased demands in a caregiving role, household and family responsibilities, and gender inequalities in work (64–66). Accordingly, to cope with mental distress, women may tend to increase their alcohol consumption (7, 67–69). This trend is unfortunately not recent. Previous data confirm that even though women usually drink less than men, gender gap in alcohol consumption is getting narrower over time (70, 71). Alcohol consumption as a coping mechanism with distress was more relevant to women's than men's drinking, especially in the past decades where women's roles have increased with more employment opportunities, and family responsibilities (71–73). Marital status seems to be a significant feature of concurrency symptoms. Being divorced, separated, or widowed compared to married people was associated with higher odds of binge drinking and distress, findings that align with previous literature (43, 61, 74–76). It has been indicated that divorced, separated, or widowed indvidiuals tend to lack social support (75, 77, 78), suffer from mental distress (77, 79, 80), loneliness and isolation (81), which in its turn can highly contribute to increased alcohol consumption and psychological distress (81, 82).

The etiology and treatment of concurrent mental health and alcohol use problems are typically more complex than either alcohol use or mental health disorders alone (33). Effective screening and early intervention are predicated on the ability to identify the populations at increased risk for concurrent disorders. Our findings indicate that population-based initiatives designed to allocate greater resources and awareness to health and gender inequities and that target the subpopulations identified would be helpful to informing the development of primary and secondary prevention and treatment programs that adopt a concurrent disorders framework. This can be done by removing barriers so that everyone including vulnerable populations, have an equal opportunity to access resources especially for clinical diagnoses and treatment options. Increasing resources for mental health treatment sessions including telehealth, and tele-mental health, especially during confinement periods, has been noted to be a promising, appropriate and feasible approach for providing mental health support during the COVID-19 pandemic (83).

Additionally, telehealth can be used to provide resources for alcohol counseling and addiction treatment, and to follow up closely with patients and give them 24/7 access to care (84). But, according to recent reports (85), a lot of work is needed to support large-scale dissemination and implementation through a multisectoral approach that has the capacity to respond to social needs, mobilize community social capital (8, 86), and provide training to social and health care providers on substance use and mental health disorders (8). These approaches can reduce health, economic and social inequity (87) that exacerbate mental illness (7) and alcohol consumption (88).

This study has several limitations. First, the study's cross-sectional nature with self-reported responses prohibited the determination of any causal relationship between substance use and emotional distress with COVID-19 risk factors. Second, response bias might have occurred from the self-reported answers. Third, survey questions were only accessible for those who have access to electronic devices and the internet, limiting the generalizability of findings. Also, this secondary data analysis was restricted to the current risk factors and questions on frequency of alcohol intake, therefore, we could not capture additional modifiable risk and protective factors associated with changes in alcohol intake, patterns of use and associated impairment, and mental health. Furthermore, future longitudinal analyses will provide a greater understanding of directionality of the observed associations, along with potentially identifying factors that mediate or moderate these associations.

## Conclusion

Our results indicate that symptoms of concurrent mental distress and alcohol use are a serious concern during the pandemic, and women and older adults, divorced, separated, or widowed, and individuals with financial worries, COVID-illness worries, and higher income groups appear to be at highest risk. As the pandemic continues to evolve, further research evaluating risk and protective factors and the long-term impact that COVID-19 has on mental health and alcohol intake is warranted to inform treatment and prevention strategies.

## Data Availability Statement

Publicly available datasets were analyzed in this study. This data can be found here: http://www.delvinia.com/solutions/askingcanadians.

## Ethics Statement

The studies involving human participants were reviewed and approved by Research Ethics Board at the Centre for Addiction and Mental Health and Research Ethics Boards of the Children's Hospital of Eastern Ontario (CHEO). The patients/participants provided their written informed consent to participate in this study.

## Author Contributions

FM: conceptualization, methodology, analysis, writing, review, and editing. HS-K: methodology, analysis, and review. BH: methodology and analysis. KC and HH: writing, review, and editing. GG: conceptualization, methodology, writing, review and editing. All authors contributed to the article and approved the submitted version.

## Conflict of Interest

The authors declare that the research was conducted in the absence of any commercial or financial relationships that could be construed as a potential conflict of interest.
